# Improved Lower Bounds of DNA Tags Based on a Modified Genetic Algorithm

**DOI:** 10.1371/journal.pone.0110640

**Published:** 2015-02-18

**Authors:** Bin Wang, Xiaopeng Wei, Jing Dong, Qiang Zhang

**Affiliations:** Key Laboratory of Advanced Design and Intelligent Computing (Dalian University), Ministry of Education, Dalian, 116622, China; Georgia Institute of Technology, UNITED STATES

## Abstract

The well-known massively parallel sequencing method is efficient and it can obtain sequence data from multiple individual samples. In order to ensure that sequencing, replication, and oligonucleotide synthesis errors do not result in tags (or barcodes) that are unrecoverable or confused, the tag sequences should be abundant and sufficiently different. Recently, many design methods have been proposed for correcting errors in data using error-correcting codes. The existing tag sets contain small tag sequences, so we used a modified genetic algorithm to improve the lower bound of the tag sets in this study. Compared with previous research, our algorithm is effective for designing sets of DNA tags. Moreover, the GC content determined by existing methods includes an imprecise range. Thus, we improved the GC content determination method to obtain tag sets that control the GC content in a more precise range. Finally, previous studies have only considered perfect self-complementarity. Thus, we considered the crossover between different tags and introduced an improved constraint into the design of tag sets.

## Introduction

In a single run, hundreds of millions of short reads can be produced by next generation sequencing instruments and this output rate will soon increase to billions of reads. Next generation sequencing is a very powerful method if relatively small DNA fragments need to be sequenced using a large number of samples. This approach requires specific sequence tags that allow the detection and identification of the address of any sequence in a mixture and its assignment back to the original sample [1–9]. During library preparation, each DNA fragment is appended with a short oligonucleotide sequence called a tag (or barcode) to deconvolve the sequencing data for each sample during data analysis. Each sample is labeled with a different tag and these DNA tags are sequenced with the DNA or RNA from the sample, either as a paired run or as a longer continuous read [2]. Since the development of next generation technologies, the sequencing accuracy has improved greatly, but sequencing errors are still inevitable. As the number of multiplexed samples increases, there is also an increased likelihood that sequencing errors in the barcodes will prevent the definitive assignment of a sequencing read to a sample, which may result in the loss of data or the transformation of one tag into another, both of which cause sample misclassification. Therefore, there is a need to develop tags that can automatically detect and correct the errors introduced during sequencing [2].

The errors often occur during the amplicon generation or library preparation processes, as well as the coupling reaction [4, 10–13]. Researchers use thermostable DNA polymerases and the polymerase chain reaction (PCR) to generate amplicons, which increases the library concentration, but errors are inevitable. Although most DNA polymerases can produce new DNA strands that contain insertion or deletion errors at low frequencies, thermostable DNA polymerases often incorporate substitution errors into DNA strands during replication [14–16]. In PCR, n-1, n-2, and n-3 congeners that contain deletion errors throughout the oligos are produced due to coupling errors [17]. Relatively expensive purification techniques can remove most of these congeners, particularly the n-2 and n-3 varieties, but some n-1 congeners remain, even with increasingly sophisticated purification methods [18]. The types of errors and the error rates vary among next generation sequencing platforms [4, 19–24]. Recently, researchers have constructed sequence tags using error correction schemes, which are more robust to synthesis, replication, and sequencing errors (i.e., minimizing crossover and loss), while also allowing the correction of certain types of errors [1–4, 12, 13, 25, 26]. Hamming codes [27] are used widely to design DNA tags. In addition, Ashlock et al. used evolutionary algorithms to design DNA tags based on the edit distance [12, 13], where they proposed greedy closure evolutionary algorithms as a modification of Conway’s lexicode algorithm. In [12], they presented a method that used a genetic algorithm to evolve controls for a greedy algorithm. Comparing with the plain lexicode algorithm, the proposed algorithm improved the lower bound of some DNA tags. In [13], they resolved the issue of the utility of the crossover operator employed in [12] for optimizing DNA error-correcting codes. They also improved the lower bounds. However, these methods did not consider specific biological characteristics when designing DNA tags, such as the GC content and perfect self-complementarity. Hamady et al. developed a set of error-correcting sequence tags, which they used to successfully track a large number of reads in a multiplex [1]. Krishnan et al. designed DNA barcodes based on BCH codes that guaranteed the correction of errors within these barcodes [2]. Bystrykh aimed to provide relatively simple, ready-made examples for use by molecular biologists whenever they need to select their own list of tags for a specific application to achieve the best possible result [3]. Faircloth et al. developed an open-source software package to validate sequence tags to ensure conformance with two-distance metrics and used this software package to evaluate several commercial and non-commercial sequence tag sets, to design several large sets of edit metric sequence tags with different lengths and degrees of error correction, and to integrate a subset of these edit metric tags into PCR primers and sequencing adapters [4]. Costea et al. proposed the DNA-based tag generator and demultiplexor (TagGD), which is a fully-customizable, rapid, and accurate software package that can generate thousands of barcodes to satisfy user-defined constraints and guarantee full demultiplexing accuracy [25]. Schober et al. proposed a simple randomized method for constructing barcodes with better error-correcting capabilities compared with previous methods [26]. Recently, Buschmann and Bystrykh adapted the Levenshtein distances by considering the DNA context where the length of the new mutated barcode in the sequence read was identified correctly [8].

Although Hamming codes can correct substitution errors, they are ineffective for insertion and deletion errors [4]. The edit distance or Levenshtein distance define the allowed operations as the removal or insertion of a single character, or the substitution of one character for another [28]. These metrics can be optimized between constituent codewords to solve the problem of insertion and deletion errors. In this study, we propose the use of a modified genetic algorithm to improve the lower bound of tag sets based on the edit distance, which is more effective for designing sets of DNA tags compared with previous methods. In existing methods, GC content is specified in a general range, which is not sufficiently precise when designing DNA tags. Thus, we used an algorithm to design DNA tag sets that are constrained within a more precise range. An improved constraint is introduced to prevent crossover between different tags, which is used to design DNA tag sets in combination with the edit distance.

## Methods

### Edit Distance

In information theory, the Hamming distance between two strings of equal length is the number of positions where the corresponding symbols differ [27]. It also measures the minimum number of substitutions required to change one string into another, or the minimum number of errors that could transform one string into another. The edit distance is a string metric used to measure the difference between two sequences [28]. Informally, the edit distance between two words is the minimum number of single character edits (insertion, deletion, or substitution) required to change one word into the other.

In the alphabet Σ = {A, C, G, T}, there exists a set *C* with a size of |C| = 4^*n*^. The code words in set C have length *n*. S is a subset of *C*. u and v are its constituent codewords, which satisfy:

τ(u,v)≥d (1)

where *d* is a positive integer and τ represents the constraint criteria (or a criterion) for DNA tags, such as the Hamming distance or edit distance [29]. In this study, τ denotes the edit distance.

### Tag-tag Edit Distance (TTE)

Tag-tag edit distance constraint: for a subset of DNA tags *S* with |S| = m (written from the 5′ to the 3′ end) and its constituent codewords *u*,*v*, *E*(*u*,*v*) denotes the edit distance between *u* and *v*. *TTE*(*u*_*i*_) denotes the minimal *E*(*u*_*i*_,*v*_*j*_) in all DNA tags and it should not be less than parameter *d*,

$$TTE(u_{i})=\underset{1\leq j\leq m,u_{i}\neq v_{j}}{\min}\left\{E\left(u_{i},v_{j}\right)\right\}\geq d$$ (2)

For example, *a* = ‘ACTG,’ *b* = ‘CAGT,’ and *c* = ‘GAGT,’ thus E(a,b) = 3, E(a,c) = 3 and E(b,c) = 1. Thus, TTE(a) = 3, TTE(b) = 1 and TTE(c) = 1. For *d* = 1, the DNA tag set includes a, b, and c, whereas for *d* = 2 or 3, the DNA tag set only includes ab or ac. This constraint is used to ensure that the edit distance of any pair of tags in the DNA tag sets are equal to or greater than d.

### GC Content

Similar melting temperatures can be obtained by ensuring that each word contains the same number of positions that are either G or C (yielding a constant GC content). Thus, the GC content constraint approximates the melting temperatures of the DNA tags and it is combined with the distance constraint. This denotes the percentage of G or C nucleotides within each DNA tag. In a previous study, the number of GC bases was denoted as Num_gc [29, 30]. The GC content is described as follows.

GC_content=Num_gcn×100% (3)

### Perfect Self-complementarity

For all pairs of *u*_*i*_ in S, *u*′ is the self-complementarity of *u*, *u*_*i*_≠*u*_*i*_′, and *i* = 1, 2, 3 … m, m is the number of DNA tags. This constraint prevents a tag in the sample from reacting with other tags in the same sample. For example, a tag ‘AATT’ is complementary to ‘AATT,’ which is the same as itself.

### Continuity

If the same base appears continuously, the structure of the DNA will become unstable. Thus, this constraint is used to control the continuous occurrence of the same base. LS(*u*_*i*_) is denoted as the length of the longest substring of *u*_*i*_. Thus, the continuity is denoted as follows:

$$Continuity=\underset{1\leq i\leq m}{Max}\left\{LS(u_{i})\right\}\leq 2$$ (4)

where *m* is the size of subset *S*. For example, u_1_ = ‘AATGC,’ u_2_ = ‘AAATTG,’ and u_3_ = ‘TCGTCA,’ thus LS(u_1_) = 2, LS(u_2_) = 3, and LS(u_3_) = 1. For the DNA tags subset {*u_1_, u_2_, u_3_*}, the continuity is equal to 3. In this study, we filtered DNA sequences if their continuity was higher than 2.

### Algorithm Design

Genetic algorithms (GAs) are adaptive heuristic search algorithms, which are based on evolutionary concepts of natural selection and genetics. GAs belong to the larger class of evolutionary algorithms, which generate solutions to optimization problems using techniques inspired by natural evolution, such as inheritance, mutation, selection, and crossover [31]. In this study, we use a modified GA to design DNA sequence tag sets based on edit distance constraints. This method improves the global search capabilities of a traditional GA. These improvements include initializing the algorithm populations in an evenly distributed manner. Unlike the random initial populations used in GAs, the individuals in the initial population of the modified GA are selected with equidistant distribution in the solution space. This operation is referred to as the evenly distributed method in the present study and it enhances the heterogeneity of the populations based on a global field. According to the number of populations, the populations are distributed evenly in the value range according to the evenly distributed method. Randomly re-initializing the populations when they satisfy certain conditions overcomes premature convergence. Population re-initialization occurs only once because increased time would decrease the rate of convergence of the algorithm. During the mutation process, we adjust the probability of a mutation operator with a dynamic method. The traditional GA adopts unique values to process the mutation operation, which could reduce the rate of convergence. The optimization problem is defined as the maximum value problem. We denote the fitness function *f*(*i*) as follows.

$$f(i)=TTE(u_{i})=\underset{1\leq j\leq m,u_{i}\neq v_{j}}{\min}\left\{E\left(u_{i},v_{j}\right)\right\}$$ (5)

The algorithm initializes DNA tags with the evenly distributed method and selects sequences that satisfy the constraint (or constraints), before generating new DNA tags using selection, crossover, and mutation operators, which finally yields the desired DNA tag sets. The pseudocode of the algorithm used to design DNA tag sets with the modified GA is as follows.

**Algorithm:** The modified GA

**Require:** Set parameters and initialize the population with an evenly distributed method.

**If** the mean of the fitness function is smaller than *f(i)*

  Randomly re-initialize the populations

**End if**

**While** the number of generations is smaller than 200 **do**

**In selection operation**

  The size of the tournament is 2 and the number of repetitions is equal to 10% of the total population in the random tournament selection

**In crossover operation**

  The three-point crossover strategy is used.

**In mutation operation**

  The probability of mutation is set dynamically.

**If** the fitness is larger than the mean of the fitness function

   its probability of mutation is 0.01

**Elseif** the fitness is equal to the mean of the fitness function

   its probability of mutation is 0.03

**Else**

   its probability of mutation is 0.3

**Endif**

  Generate

**EndWhile**

For example, we generate the initial population P = {p_1_,p_2_,…,p_k_} and result set S = {s_1_,s_2_,…, s_m_}. Next, we calculate the fitness, i.e., $f(i)=TTE(p_{i})=\underset{1\leq j\leq m,p_{i}\neq s_{j}}{\min}\left\{E\left(p_{i},s_{j}\right)\right\}$. The first element of the result set is selected randomly from the initial population. If f(i)≥d in the population, the DNA tag *p*_*i*_ is appended to the result set as a new element. Throughout the evolution of the modified GA, the number of elements in the result set increases until the evolution process is complete.

We compared the performance of our algorithms with previous methods and we obtained better results based on many different combinatorial constraints [29, 30]. Thus, our algorithm is suitable for designing DNA tag sets that satisfy the edit distance constraint. In the studies reported by Ashlock et al. [12, 13], greedy closure evolutionary algorithms were proposed as a modification of Conway’s lexicode algorithm. In the present study, we use two sets to implement our algorithm: one to ensure the completion evolution and another for storing the results. The fitness of the chromosomes in the first set is determined by the chromosomes in the other set.

## Results

The parameters of the modified GA used in our example were as follows: population size = 500, crossover rate = 0.45, initial probability of a mutation = 0.01. To control the runtime of the algorithm, the number of generations was set to 200. To increase the reliability of our experimental results, we performed 100 experiments for each value and we report the maximum values obtained in these experiments. In the tables, *d* is the edit distance and *n* is the length of the DNA tags.

Note that the number of generations was set to 200 because of the runtime of the algorithm. For small sets, only a few minutes were required to run 200 generations. However, hundreds of hours were required for large sets, such as n = 10, d = 3. Furthermore, the runtime increased rapidly as the number of generations increased. Given the time consumption and the results required, the number of generations was set to 200.

### Comparison with the Method of Faircloth et al. [4]

Faircloth et al. designed several large tag sets that comprised 4–10 nucleotides in length with a minimum edit distance of three. In their study, they used an improved lexicode algorithm to design the DNA tag sets. The GC content was in the range of 40% ≤ GC ≤60%. The DNA sequences were filtered if their continuity was higher than 2. The results obtained using this method are shown in Table 1.

**Table 1:** Results obtained by Faircloth et al.

n\d	3	4	5	6	7	8	9
4	7						
5	25	7					
6	61	15	5				
7	211	41	11	4			
8	531	103	24	8	3		
9	1936	301	62	18	6	3	
10	7198	971	164	40	13	5	3

### Improving the Lower Bound of DNA Tag Sets

Table 1 shows the results obtained using the method of Faircloth et al. [4], which satisfied the edit distance, GC content, and continuity constraints. For n = 10 and d = 7, the value given in the Table 2 of reference [4] was 14, whereas this value was 13 in S1 in the present study. Thus, we used 13 as the value. We designed DNA tag sets that satisfied the same combinatorial constraints and the results are given in Table 2, where the bold numbers denote the results that are better than or equal to the corresponding results in Table 1. A comparison of Table 1 and Table 2 shows that our method could improve the lower bounds that satisfied the combinational constraints, as well as further reducing the range of the bounds for the DNA tag sets. Thus, our algorithm is more effective for designing DNA tag sets. Although our algorithm is not ideal for large tag sets, it is effective for small tags sets. In future research, we will improve our algorithm to overcome this problem.

**Table 2:** Results obtained using our method.

n\d	3	4	5	6	7	8	9
4	**10**						
5	**25**	**8**					
6	49	**15**	**6**				
7	133	34	**11**	**5**			
8	296	73	23	**9**	**4**		
9	876	180	45	15	**7**	**4**	
10	1863	399	91	28	12	**6**	**3**

### Improved GC Content Constraint

Considering the combinational constraints described above, the range of the GC content was controlled to be between 40% and 60%, which is the rough range used for the design of DNA tags. It is well known that the GC content affects the thermodynamic properties of DNA tags, i.e., the melting temperature (Tm). During the design of the DNA tags, we tried to ensure that the GC content had a uniform value to maintain similar thermodynamic properties. Thus, we developed an improved method for controlling the GC content constraint. Let the number of G or C be equal to n2n×100%, and ⌊A⌋ rounds the elements of A to the nearest integers that are less than or equal to A.

According to Faircloth et al., the range of the GC content was controlled to be between 40% and 60%. Furthermore, other studies have considered the same approach to this problem [1–3, 5, 25]. Table 3 shows the DNA tag sets we designed to satisfy the edit distance, improved GC content, perfect self-complementarity, and continuity constraints.

**Table 3:** Results obtained using the improved GC content.

n\d	3	4	5	6	7	8	9
4	10						
5	18	7					
6	49	15	6				
7	109	28	10	4			
8	296	73	23	9	4		
9	724	168	41	14	6	3	
10	1651	373	86	26	10	5	3

The multiple hybridization reaction involves distinct DNA sequences, thus the Tms of the duplexes created in each reaction should be within a narrow range. The hybridization reaction is more stable when the range is smaller [32, 33]. In this study, we propose the use of a Tm gap to evaluate the quality of DNA tag sets, which is denoted as follows:

Tm_gap=|Max_Tm−Min_Tm|, (6)

where Max_Tm is the maximum Tm in a DNA tag set and Min_Tm is the minimum Tm in the same DNA tag set. Obviously, the quality of a DNA tag set is better if the Tm gap is smaller.

In this study, the Tm value of each DNA tag was calculated using a nearest-neighbor model [34]. Note that the improved GC content constraint is the same as the original GC content constraint when the length of the DNA tag is equal to 4, 6, and 8, because the GC content is equal to 50% with these lengths. Table 4 shows the Tm gaps for the DNA tag sets in Table 1 and Table 5 shows those for Table 3, where the lengths of the DNA tags were equal to 5, 7, 9, and 10. In Table 5, the results are better than the corresponding results in Table 4. This demonstrates that the improved GC content constraint is better than the original GC content constraint.

**Table 4:** Tm gap using the method of Faircloth et al.

n\d	3	4	5	6	7	8	9
5	22.6896	22.8938					
7	21.5792	17.4198	15.4809	11.8720			
9	19.0211	18.1603	16.2628	15.8941	10.1666	4.4620	
10	25.3735	23.3012	19.4685	16.2235	12.0353	9.1415	10.0362

**Table 5:** Tm gap using the improved GC content.

n\d	3	4	5	6	7	8	9
5	11.4904	10.0252					
7	11.9657	12.5683	10.7165	6.0446			
9	14.4468	13.0727	10.8129	10.9390	4.9747	2.8215	
10	13.9062	12.5916	10.1544	8.0374	7.7544	4.3199	2.7314

### Perfect Complementarity

During next generation sequencing, each DNA fragment is appended with a short oligonucleotide sequence called a tag (or barcode) to deconvolve the sequencing data for each sample during data analysis. Each short oligonucleotide from the same sample is labeled with the same tag, whereas different samples are labeled with different tags. According to the constraint of perfect self-complementarity, this constraint prevents the tag of sample from reacting with other tags from the same sample. However, it cannot prevent a tag from reacting with other tags from different samples. For example, the sequences ‘GTCAA’ and ‘TTGAC’ are included in the DNA tag sets reported by Faircloth et al. for n = 5, d = 3. These sequences are mutually complementary because ‘GTCAA’ is the perfect complement to ‘TTGAC,’ and vice versa. Using these tags will increase the likelihood of error reactions between tags from different samples. Thus, we propose an improved constraint to control the error reaction.

For all pairs of *u*_*i*_ in S, *v*_*j*_′ is the complement of *v*, *u*_*i*_ ≠*v*_*j*_′, i,j = 1,2,3…n. This constraint prevents the tag of a sample from reacting with other tags from different samples. Table 6 shows the results obtained using the method that satisfies the edit distance, the proposed GC content, and the proposed perfect complementarity and continuity constraints. Note that the combinatorial constraints do not include perfect self-complementarity. This is because perfect self-complementarity is included in the proposed perfect complementarity constraint when i = j.

**Table 6:** Results obtained using the improved perfect complementarity constraint.

n\d	3	4	5	6	7	8	9
4	9						
5	14	7					
6	45	15	6				
7	103	26	10	4			
8	274	73	23	9	4		
9	688	168	41	14	6	4	
10	1579	371	86	26	10	5	3

The results in Table 6 are derived from Table 3, although some DNA tags that do not satisfy the perfect complementarity condition have been deleted. Note that the tags where n = 4, d = 3 were all deleted because these tags did not satisfy the perfect complementarity condition. Thus, we used our algorithm to redesign these tags. The numbers of other DNA tags were not changed significantly by the improved conditions.

## Discussion

Compared with the method of Faircloth et al., some lower bounds were improved according to our results. Faircloth et al. modified the lexicode algorithm to design DNA tags sets. In order to enhance the running speed of their algorithm, they only retained the DNA tags with the maximum count based on comparisons. However, the retained DNA tags could not be the codewords with the maximum count in the DNA tag sets. Thus, this approach is not very effective for some sets.

Our results can also be improved because the population size and the number of generations increase with the proposed algorithm, but they are not ideal for large tag sets. In future research, we will improve our algorithm to overcome this problem.

Tables 4 and 5 show that the Tm gap decreased as the edit distance increased, apart from a few sets. There may be a strict relationship between the increase in the edit distance and the decrease in the Tm gap, which we will investigate in future research.

## Conclusions

Our novel method uses a modified GA to design DNA tag (or barcode) sets based on combinatorial constraints. Compared with previous studies, our algorithm improves the lower bounds of DNA tag sets, which satisfy the edit distance, original GC content, perfect self-complementarity, and continuity constraints. We found that our algorithm was highly effective for designing DNA tag sets. The original GC content was required to lie within an approximate range in previous studies because it affects the thermodynamic properties when designing DNA tags, thus we improved the GC content by controlling it within a more precise range. Finally, we combined the improved GC content with the edit distance, perfect self-complementarity, and continuity constraints to design DNA tag sets. To prevent tags from reacting with other tags from different samples during next generation sequencing, we propose the introduction of an improved perfect self-complementarity constraint in the design of DNA tag sets, which can be combined with the edit distance, improved GC content, and continuity constraints.

## Supporting Information

S1 File The DNA sequences are included in Table 2, Table 3 and Table 6. (ZIP)
