# Correction: Improved Lower Bounds of DNA Tags Based on a Modified Genetic Algorithm

**DOI:** 10.1371/journal.pone.0130784

**Published:** 2015-06-23

**Authors:** Bin Wang, Xiaopeng Wei, Jing Dong, Qiang Zhang

In “Improved Lower Bounds of DNA Tags Based on a Modified Genetic Algorithm,” by Bin Wang *et al*, there were several instances of text overlap with published sources. The authors did include references for these sources, and in some cases provided in-text citations. They have now been informed of appropriate ways to discuss and attribute others’ work and expressions in scientific literature.

The authors apologize for the text overlap with, and incomplete attribution of expressions to, published works in this *PLOS ONE* article, and the first author apologizes to all the other authors for his negligence. This Correction is being published to give appropriate attribution to the sources of expressions and text overlap. The following blocks of text are derived from—and in several cases comprised verbatim of—text in the indicated sources, although sources were cited in some instances. In each case, the section of the article is provided to help readers locate the overlapping text within the published article.

*from* **Faircloth, B. C. and Glenn, T. C. (2012). Not All Sequence Tags Are Created Equal: Designing and Validating Sequence Identification Tags Robust to Indels. *PLOS ONE* 7: e42542. DOI:** 10.1371/journal.pone.0042543 **(http://journals.plos.org/plosone/article?id=10.1371/journal.pone.0042543)**

*This publication is cited as Reference 4.

- Abstract:
  - “obtain sequence data from multiple individual samples. In order to ensure that sequencing, replication, and oligonucleotide synthesis errors do not result in tags (or barcodes) that are unrecoverable or confused, the tag sequences should be abundant and sufficiently different.”
- Introduction:
  - “often occur during the amplicon generation or library preparation processes, as well as the coupling reaction [4,10–13]. Researchers use thermostable DNA polymerases and the polymerase chain reaction (PCR) to generate amplicons, which increases the library concentration, but errors are inevitable. Although most DNA polymerases can produce new DNA strands that contain insertion or deletion errors at low frequencies, thermostable DNA polymerases often incorporate substitution errors into DNA strands during replication [14–16]. In PCR, n-1, n-2, and n-3 congeners that contain deletion errors throughout the oligos are produced due to coupling errors [17]. Relatively expensive purification techniques can remove most of these congeners, particularly the n-2 and n-3 varieties, but some n-1 congeners remain, even with increasingly sophisticated purification methods [18]. The types of errors and the error rates vary”
  - “are more robust to synthesis, replication, and sequencing errors (i.e., minimizing crossover and loss), while also allowing the correction of certain types of errors [1–4, 12, 13, 25, 26].”
  - “a set of error-correcting sequence tags, which they used to successfully track a large number of reads in a multiplex [1]”
  - “developed an open-source software package to validate sequence tags to ensure conformance with two-distance metrics and used this software package to evaluate several commercial and non-commercial sequence tag sets, to design several large sets of edit metric sequence tags with different lengths and degrees of error correction, and to integrate a subset of these edit metric tags into PCR primers and sequencing adapters [4]”

*from* **Krishnan, A. R., Sweeney, M., Vasic, J., Galbraith, D.W., and Vasic, B. (2011). Barcodes for DNA sequencing with guaranteed error correction capability. *Electronics Letters* 47: 236–237. (http://www.crossref.org/iPage?doi=10.1049%2Fel.2010.3546)**

*This publication is cited as Reference 2.

- Introduction:
  - “short oligonucleotide sequence called a tag (or barcode) to deconvolve the sequencing data for each sample during data analysis. Each sample is labeled with a different tag and these DNA tags are sequenced with the DNA or RNA from the sample, either as a paired run or as a longer continuous read [2]. Since the development of next generation technologies, the sequencing accuracy has improved greatly, but sequencing errors are still inevitable. As the number of multiplexed samples increases, there is also an increased likelihood that sequencing errors in the barcodes will prevent the definitive assignment of a sequencing read to a sample, which may result in the loss of data or the transformation of one tag into another, both of which cause sample misclassification. Therefore, there is a need to develop tags that can automatically detect and correct the errors introduced during sequencing [2]”
- Perfect Complementarity:
  - “to deconvolve the sequencing data for each sample during data analysis.”

*from* **Bystrykh, L. V. (2012). Generalized DNA Barcode Design Based on Hamming Codes. *PLOS ONE* 7: e36852. DOI:** 10.1371/journal.pone.0036852 **(http://journals.plos.org/plosone/article?id=10.1371/journal.pone.0036852)**

*This publication is cited as Reference 3.

- Introduction:
  - “sequencing is a very powerful method if relatively small DNA fragments need to be sequenced using a large number of samples. This approach requires specific sequence tags that allow the detection and identification of the address of any sequence in a mixture and its assignment back to the original sample”
  - “to provide relatively simple, ready-made examples for use by molecular biologists whenever they need to select their own list of tags for a specific application to achieve the best possible result [3]”

*from* **Zhang, Q., Wang, B., Wei, X. and Zhou, C. (2013). A Novel Constraint for Thermodynamically Designing DNA Sequences. *PLOS ONE* 8: e72180. DOI:** 10.1371/journal.pone.0072180 **(http://journals.plos.org/plosone/article?id=10.1371/journal.pone.0072180)**

*This publication is by the same author as the Corrected PLOS ONE article, and is cited as Reference 29.

- Algorithm Design:
  - “Genetic algorithms (GAs) are adaptive heuristic search algorithms, which are based on evolutionary concepts of natural selection and genetics.”
  - “populations based on a global field. According to the number of populations, the populations are distributed evenly in the value range according to the evenly distributed method. Randomly re-initializing the populations when they satisfy certain conditions overcomes premature convergence. Population re-initialization occurs only once because increased time would decrease the rate of convergence of the algorithm. During the mutation process, we adjust the probability of a mutation operator with a dynamic method. The traditional GA adopts unique values to process the mutation operation, which could reduce the rate of convergence. The optimization problem is defined as the maximum value problem. We denote the fitness function f(i)“
  - “The algorithm initializes DNA tags with the evenly distributed method and selects sequences that satisfy the constraint (or constraints), before generating new DNA tags using selection, crossover, and mutation operators, which finally yields the desired DNA tag sets.”

Some other elements of the Algorithm Design were also published in Zhang et al, 2013.

- Results:
  - “The parameters of the modified GA used in our example were as follows: population size = 500, crossover rate = 0.45, initial probability of a mutation = 0.01. To control the runtime of the algorithm, the number of generations was set to 200. To increase the reliability of our experimental results, we performed 100 experiments for each value and we report the maximum values obtained in these experiments. In the tables, d is the edit distance and n is the length of the DNA tags.”
